# Influence of the 14-alkoxy group and the substitution in position 5 in *N*-methyl-14-alkoxymorphinan-6-ones on *in vitro* and *in vivo* pharmacological activities

**DOI:** 10.1186/2050-6511-13-S1-A33

**Published:** 2012-09-17

**Authors:** Tanila Ben Haddou, Roberta Lattanzi, Lucia Negri, Helmut Schmidhammer, Mariana Spetea

**Affiliations:** 1 Department of Pharmaceutical Chemistry, Institute of Pharmacy and Center for Molecular Biosciences, University of Innsbruck, 6020, Innsbruck, Austria; 2 Department of Human Physiology and Pharmacology, University ‘La Sapienza’, 00185 Rome, Italy

## Background

Opioid analgesics are the cornerstone drugs for the treatment of moderate-to-severe pain. Morphine and other analgesics like fentanyl, oxycodone and oxymorphone activate the µ opioid (MOP) receptor, the main type targeted for pharmacotherapy of pain. These drugs share the same pharmacological profiles including severe adverse effects such as respiratory depression, constipation, tolerance and physical dependence. Chemical approaches towards the identification of novel MOP analgesics with reduced side effects include structural modifications of morphinan-6-ones in key positions that are important for binding, selectivity, potency and efficacy at opioid receptors. A representative example is the development of the 14-*O*-methyl-substituted derivative of the clinically used MOP analgesic oxymorphone, namely 14-*O*-methyloxymorphone, and its 5-methyl-substituted analogue, 14-methoxymetopon. The focus of the present work is on structure-activity relationship (SAR) studies and *in vitro* and *in vivo* pharmacological investigations on a series of opioid ligands differently substituted in positions 5 and 14 of the morphinan skeleton.

## Methods

Radioligand binding assays were performed using rodent brain membranes. Mouse vas deferens (MVD) and guinea-pig ileum (GPI) bioassays, and [^35^S]GTPγS functional assays with Chinese hamster ovary (CHO) cells expressing human opioid receptors were used to assess opioid agonism. Antinociceptive properties were established using hot-plate and writhing tests in mice after subcutaneous (s.c.) administration.

## Results

Binding studies showed that all derivatives display affinities in the subnanomolar range at the MOP receptor and were MOP receptor-selective. In smooth muscle preparations and CHO cells transfected with MOP receptors they behaved as potent agonists. The differently substituted *N*-methylmorphinan-6-ones produced marked antinociceptive effects in mice when given s.c., being several-fold more potent than morphine. On the basis of the SAR that has emerged, certain modifications in the substitution pattern, e.g. introduction of an alkyl or arylalkyl group in position 14 and/or in position 5, result in interesting alterations in opioid activity by influencing the pharmacological properties of ligands interacting with opioid receptors. Analysis of the *in vitro* and *in vivo* opioid profile for this series of 14-alkoxymorphinans leads to an improved understanding of the relationship between affinity and/or selectivity for opioid receptors, agonist activity, antinociceptive potency and the nature of substituents in morphinans.

## Conclusions

These results represent a useful and valuable outcome for the design and optimization of existing structural templates increasing the chance of identifying clinically useful analgesics for superior management of pain.

